# SARS-CoV-2 Transmission in Patients With Cancer at a Tertiary Care Hospital in Wuhan, China

**DOI:** 10.1001/jamaoncol.2020.0980

**Published:** 2020-03-25

**Authors:** Jing Yu, Wen Ouyang, Melvin L. K. Chua, Conghua Xie

**Affiliations:** 1 Department of Radiation and Medical Oncology, Zhongnan Hospital of Wuhan University, Wuhan, Hubei, People’s Republic of China; 2 Hubei Key Laboratory of Tumor Biological Behaviors, Zhongnan Hospital of Wuhan University, Wuhan, Hubei, People’s Republic of China; 3 Hubei Cancer Clinical Study Center, Zhongnan Hospital of Wuhan University, Wuhan, Hubei, People’s Republic of China; 4 Division of Radiation Oncology, Division of Medical Sciences, National Cancer Centre Singapore, Singapore; 5 Oncology Academic Programme, Duke-NUS Medical School, Singapore

## Abstract

This cross-sectional study reviews the medical records of 1524 patients with cancer treated at a single tertiary care hospital in Wuhan, China, to evaluate the characteristics associated with transmission of the SARS-CoV-2 virus.

In December 2019, an outbreak of 2019 novel coronavirus disease (COVID-19) occurred in Wuhan, Hubei, which has been linked to the severe adult respiratory syndrome coronavirus 2 (SARS-CoV-2). It is characterized by rapid human-to-human transmission from droplet contamination.^1,2^ A report of 138 hospitalized patients from a single institution (Zhongnan Hospital of Wuhan University) indicated that hospital-acquired transmission accounted for 41.3% of these admitted patients, thus implicating the hospital environment as a source of spread of the virus.^3^ Patients with cancer are often recalled to the hospital for treatment and monitoring, and hence, they may be at risk of contracting COVID-19. Moreover, cancer treatments such as chemotherapy and radiotherapy are immunosuppressive. Here, we report the incidence and outcomes of SARS-CoV-2 infection in cancer patients who were treated at a tertiary cancer institution in Wuhan.

## Methods

We reviewed the medical records, including demographic, clinical, and treatment data of 1524 patients with cancer who were admitted to the Department of Radiation and Medical Oncology, Zhongnan Hospital of Wuhan University, from December 30, 2019, to February 17, 2020 (data cutoff date). COVID-19 pneumonia was diagnosed based on the updated COVID-19 Diagnostic Criteria, 5th Edition (Supplement). Outcomes of COVID-19 among patients with cancer were reported.

This retrospective study was approved by the Zhongnan Hospital of Wuhan University ethics committee (2020039). Waiver of informed consent was approved for the aggregated data; verbal informed consent was obtained from the living patients with COVID-19.

## Results

We estimated the infection rate of SARS-CoV-2 in patients with cancer from our single institution at 0.79% (12 of 1524 patients; 95% CI, 0.3%-1.2%). This was higher than the cumulative incidence of all diagnosed COVID-19 cases that was reported in the city of Wuhan over the same time period (0.37%; 41 152 of 11 081 000 cases; data cutoff on February 17, 2020). Clinical details on the cancer diagnoses and treatment history are summarized in Table 1. The median age of infected patients was 66 years (range, 48 to 78 years); 8 of 12 patients (66.7%) were older than 60 years. Seven of 12 (58.3%) patients had non–small cell lung carcinoma (NSCLC). Five (41.7%) were being treated with either chemotherapy with or without immunotherapy (n = 3) or radiotherapy (n = 2). Three patients (25.0%) developed SARS; 1 patient required intensive-level care. As of March 10, 2020, 6 patients (50.0%) had been discharged, whereas 3 deaths (25.0%) were recorded.

**Table 1.** Clinical Characteristics and Outcomes of Patients With Cancer With SARS-CoV-2 Infection

Patient No.	Sex	PS	Cancer diagnosis	Phase of cancer treatment	Time between onset of symptoms and diagnosis, d	Fever	Dyspnea	Cough	CT diagnosis	SARS-COV-2 RT-PCR assay	Severe COVID-19	Hospitalization status	Duration of hospitalization, d	Survival status^a^
1	Male	1	NSCLC	Concurrent EGFR TKI (osimertinib [80 mg/d]) with concurrent thoracic radiotherapy (27 Gy in 9 fr)	8	Yes	No	No	Positive	Positive	No	Discharged	19	Alive
2	Male	1	NSCLC	W5 of chemoradiotherapy (chemotherapy regime: pemetrexed [500 mg/m^2^], cisplatin [75 mg/m^2^] q3W; radiotherapy (54 Gy in 27 fr)	1	Yes	Yes	Yes	Positive	Negative	Yes	Discharged	31	Dead
3	Female	3	NSCLC	Admitted for pleural effusion; best supportive care	0	Yes	Yes	Yes	Positive	Negative	No	Discharged	13	Alive
4	Male	1	NSCLC	Follow-up at 2 y postchemoradiotherapy; no evidence of disease relapse	0	Yes	Yes	Yes	Positive	Negative	Yes	Discharged	2	Dead
5	Male	1	NSCLC	After first cycle (docetaxel, [75 mg/m^2^], cisplatin [75 mg/m^2^], sintilimab, [200 mg])	0	Yes	No	No	Positive	Negative	No	Discharged	12	Alive
6	Male	1	NSCLC	After third cycle (pemetrexed [500 mg/m^2^], carboplatin [AUC5], pembrolizumab [200 mg])	0	Yes	No	No	Positive	Negative	No	Discharged	32	Alive
7	Male	1	NSCLC	Surgery (April 23, 2019), after 4 cycles adjuvant chemotherapy (docetaxel [75 mg/m^2^], cisplatin [75 mg/m^2^])	0	Yes	No	No	Positive	Negative	No	Discharged	8	Alive
8	Male	2	Rectal cancer	Best supportive care	0	Yes	No	No	Positive	Negative	No	Inpatient	48	Alive
9	Male	1	Colon cancer	Newly diagnosed; treatment yet to commence	23	Yes	No	No	Negative	Positive	No	Inpatient	26	Alive
10	Male	3	Pancreatic cancer	Best supportive care	0	Yes	No	No	Positive	Negative	Yes	Discharged	1	Dead
11	Female	1	Breast cancer	Adjuvant radiotherapy (42 Gy in 21 fr)	2	Yes	No	No	Positive	Positive	No	Discharged	27	Alive
12	Male	2	Urothelial cancer	Best supportive care	6	Yes	No	No	Not performed	Positive	No	Inpatient	22	Alive

Abbreviations: AUC5, area under the curve 5; COVID-19, 2019 novel coronavirus disease; CT, computed tomography; EGFR, epidermal growth factor receptor; fr, radiotherapy fraction; NSCLC, non–small cell lung cancer; PS, performance status; q3w, every 3 weeks; RT-PCR, real-time quantitative polymerase chain reaction; SARS-CoV-2, severe acute respiratory syndrome coronavirus 2; TKI, tyrosine kinase inhibitor.

^a^ Survival and hospitalization status were updated as of March 10, 2020.

We also interrogated the association of SARS-Cov-2 infection with age and concurrent NSCLC diagnosis. Of the 1524 patients with cancer who were screened, 228 had NSCLC. We found that patients with NSCLC older than 60 years had a higher incidence of COVID-19 than those aged 60 years or younger (4.3% vs 1.8%) (Table 2).

**Table 2.** COVID-19 Pneumonia in Patients With NSCLC of Different Age Groups Treated at the Zhongnan Hospital of Wuhan University

Age, y	No. (%)	
	Total No. of patients with NSCLC (n = 228)	Patients with NSCLC with COVID-19 (n = 7)
≤60	111 (48.7)	2 of 111(1.8)
>60	117 (51.3)	5 of 117 (4.3)

Abbreviations: COVID-19, 2019 novel coronavirus disease; NSCLC, non–small cell lung cancer.

## Discussion

It is hypothesized that patients with cancer may be susceptible to an infection during a viral epidemic owing to their immunocompromised status.^4^ This study highlights the following observations: patients with cancer from the epicenter of a viral epidemic harbored a higher risk of SARS-CoV-2 infection (OR, 2.31; 95% CI, 1.89-3.02) compared with the community. However, fewer than half of these infected patients were undergoing active treatment for their cancers. Next, we observed that older patients (>60 years) and patients with NSCLC may be at risk of COVID-19. Nonetheless, a population study of 1099 patients with COVID-19 did not indicate that age was associated with susceptibility to infection.^5^ A larger sample size in patients with cancer will resolve these potential associations. Finally, our findings imply that hospital admission and recurrent hospital visits are potential risk factors for SARS-CoV-2 infection.

We propose that aggressive measures be undertaken to reduce frequency of hospital visits of patients with cancer during a viral epidemic going forward. For patients who require treatment, proper isolation protocols must be in place to mitigate the risk of SARS-CoV-2 infection.

## Notes

Supplement. eMethods.
