# Supplementary material for: SARS-CoV-2 Transmission in Patients With Cancer at a Tertiary Care Hospital in Wuhan, China
Source: JAMA Oncol. 2020 Mar 25;6(7):1108–10. doi: 10.1001/jamaoncol.2020.0980 (PMC7097836; doi:10.1001/jamaoncol.2020.0980)
Supplement: Supplement. — eMethods. [file jamaoncol-6-1108-s001.pdf]

Supplementary Online Content

Yu J, Ouyang W, Chua MLK, Xie C. SARS-CoV-2 Transmission in Patients With Cancer at a Tertiary Care Hospital in Wuhan, China. Published online March 25, 2020. *JAMA Oncology*.
doi:10.1001/jamaoncol.2020.0980

eMethods: 2019 Novel Coronavirus Disease (COVID-19) Diagnostic criteria 5th edition (5 Feb 2020)

This supplementary material has been provided by the authors to give readers additional information about their work.

© 2020 Yu J et al. JAMA Oncology.

eMethods

2019 Novel Coronavirus Disease (COVID-19) Diagnostic criteria 5th edition (5 Feb 2020)

Other provinces except Hubei province

1. **Suspected cases by clinical criteria**
   Comprehensive screening of the following epidemiological information and clinical manifestations:
   1) Epidemiological information
      a) Cases reporting a history of travel to or reside in Wuhan city and surrounding areas, or other communities with reported cases within 14 days prior to onset of illness.
      b) Cases have been exposed to patients with COVID-19 (confirmed by laboratory diagnosis of SARS-CoV-2 RNA detection) within 14 days prior to onset of illness.
      c) Cases have been exposed to patients with fever or respiratory symptoms from Wuhan city and surrounding areas, or other communities with reported cases within 14 days prior to onset of illness.
      d) Has contact history with a cluster.
   2) Clinical manifestations
      a) Fever and / or respiratory symptoms
      b) Computed tomography (CT) changes for viral pneumonia
      c) Normal or below normal absolute leukocyte count, or low absolute lymphocyte count below normal range in the early stages of the disease.

   Diagnosis of COVID-19 is made if 1) cases report any one of above epidemiological information and harbor any two of the clinical manifestations, or 2) cases without the above epidemiological information, but harbor all of the above clinical manifestations.

2. **Laboratory confirmed cases**
   Suspected cases with any one of the following:
   1) Respiratory tract or blood specimen test positive for SARS-CoV-2 by real-time reverse transcriptase polymerase chain reaction (RT-PCR) assay.
   2) Respiratory tract or blood specimen showing high homology with SARS-CoV-2 by genetic sequencing.

Hubei province

1. **Suspected cases**
   Comprehensive screening of the following epidemiological information and clinical manifestations:
   1) Epidemiological information
      a) Cases reporting a history of travel to or reside in Wuhan city and surrounding areas, or other communities with reported cases within 14 days prior to onset of illness.
      b) Cases have been exposed to patients with COVID-19 (confirmed by laboratory diagnosis of SARS-CoV-2 RNA detection) within 14 days prior to onset of illness.
      c) Cases have been exposed to patients with fever or respiratory symptoms from Wuhan city and surrounding areas, or other communities with reported cases within 14 days prior to onset of illness.
      d) Has contact history with a cluster.
   2) Clinical manifestations

© 2020 Yu J et al. JAMA Oncology.

a) Fever and / or respiratory symptoms
b) Normal or below normal absolute leukocyte count, or low absolute lymphocyte count below normal range in the early stages of the disease.

Diagnosis of COVID-19 is made if 1) cases report any one of above epidemiological information and harbor any two of the clinical manifestations, or 2) cases without the above epidemiological information, but harbor any two of clinical manifestations

2. Clinically diagnosed cases

CT chest changes that are consistent with atypical viral pneumonia.

3. Laboratory confirmed cases

Suspected cases or clinically diagnosed cases with any one of the following:

1) Respiratory tract or blood specimen test positive for SARS-CoV-2 by RT-PCR assay.
2) Respiratory tract or blood specimen showing high homology with SARS-CoV-2 by genetic sequencing.

© 2020 Yu J et al. JAMA Oncology.
